# Case of Rupture of the Larynx, from a Blow on the Pomum Adami, Cured in Two Weeks

**Published:** 1838-04-25

**Authors:** 


### *Case of Rupture of the Larynx, from a blow on the Pomum Adami, cured in two weeks.*

F. N——, aged 45 years, a watchman, whilst attempting to arrest a man in the neighborhood of the river, on the night of the 19th of October, 1837, was knocked down and struck on the throat by a large piece of coal. He was seen immediately by a physician, who found him nearly strangled, unable to speak, and with constant spasm, whenever he attempted to speak or swallow. He was bled freely, and sent to the hospital, eighteen hours after the accident.

*October* 20th. The throat much swelled, and inflamed externally; fauces slightly reddened; aphonia complete; breathing stertorous; barely able to whisper, and swallows with great difficulty. The cartilages of the larynx are loose and crepitant, the *thyroid* separated and moveable, one on the other. Examination of the larynx causes violent gagging; ordered sixty leeches to the outside of the throat, and warm cloths afterwards, to promote the bleeding. Gruel and tea for diet.

*October* 21st. Rested well; voice somewhat stronger; less pain in the throat; swallows rather better; ordered the same number of leeches; injection to bowels, and other treatment continued.

*October* 23d. Voice quite audible, though very hoarse; swallows well; no pain on slight pressure on the larynx; sitting up in bed; has swallowed but little since his admission. Ordered weak broth for diet.

*October* 24th. Voice gradually returning; ordered blister to the throat, to be followed by a poultice.

*October* 26th. Walking about; union of cartilages quite firm; voice improving; blister repeated; treatment continued; brown mixture for cough, which troubles him slightly.

*October* 28th. Voice nearly well, though hoarse; swallows as well as ever; cartilages united entirely.

*November* 4th. Patient discharged; voice strong; no motion in the cartilages; slight hoarseness.

*December* 15th. The patient was seen to-day; he is able to attend to his duties; speaks clearly; but is unable to call the hour, without some difficulty; has been free from pain since he was discharged.

